# Mobilization and deposition of plastic along an estuarine bank during tidal cycles

**DOI:** 10.1016/j.heliyon.2025.e42026

**Published:** 2025-01-21

**Authors:** Rosa Sawan, Périne Doyen, Guillaume Veillet, Florence Viudes, Céline Mahfouz, Rachid Amara

**Affiliations:** a Univ. Littoral Côte d’Opale, CNRS, IRD, Univ. Lille, UMR 8187 - LOG – Laboratoire d’Océanologie et de Géosciences, F-62930, Wimereux, France; b National Center for Marine Sciences, CNRS-L, Beirut, Lebanon; c Univ. Littoral Côte d’Opale, UMRt 1158 BioEcoAgro, USC ANSES, INRAe, Univ. Artois, Univ. Lille, Univ. Picardie Jules Verne, Univ. Liège, Junia, 62200, Boulogne-sur-Mer, France

**Keywords:** Mobilization, Plastic deposition, Estuary, Environmental conditions, Substrate

## Abstract

Estuaries represent a transitional environment between continental and marine areas. Limited studies have evaluated how these complex systems contribute to plastic pollution dynamics at this interface. Here, an *in situ* experimental study was conducted in the Slack estuary, a small macrotidal estuarine system in northern France, to investigate the mobilization and deposition of plastic debris on an estuarine bank at a daily basis during six complete tidal cycles. To achieve this, plastics (macro, meso and microplastics with size ≥3 mm) of different composition and shape were manually deposited along an estuary bank on three different substrates: vegetation, gravel, and sand. The experimental design aimed to explore the complexity of the mobilization and deposition of plastic debris with regard to hydro-meteorological factors, types of substrates, size and shape of plastics. Results showed that tidal cycles played a significant role in plastic mobilization and deposition on the estuary bank. However, the nature of the substrate directly impacted the mobilization and deposition of plastics and the effect of wind may be particularly important for the deposition of allochthonous plastics. Most plastics (around 94 %, 37.9 ± 1.5 plastics/m^2^) were found to be mobilized after a complete tidal cycle while an average of 3.33 ± 1.8 plastics/m^2^ was deposited during the same period. Results suggested that in small macrotidal estuaries, the daily net retention is very limited since most plastics were mobilized after a tidal cycle. However, in vegetated substrate, the daily net retention can be 2 to 3 times higher than in other types of substrates (gravel and sand) highlighting the potential of dense vegetation to serve as a retention area for plastic waste.

## Highlights

• About 94 % of manually deposited plastics were mobilized after a full tidal cycle.
• Average of 3.33 ± 1.8 plastics/m^2^ deposited, accumulated after a full tidal cycle.
• Daily net retention by vegetation can be 2 to 3 times higher than other substrates.
• The effect of wind was noticeable in the deposition of new plastics.

## Graphical abstract

**Figure:**
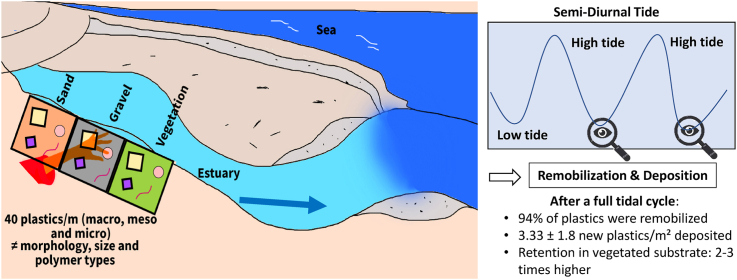

## 1 Introduction

Plastic pollution is a growing global problem that has recently gained scientific attention due to its detrimental effects at ecological, economic and societal scales [1,2]. The global mismanagement of plastic waste has contributed to this environmental crisis where almost 80 % of plastic waste is introduced into the ocean from land-based sources [3]. Rivers are one of the main pathways for the transport of plastics from the land to the sea [4]. The quantities of plastic discharged into the sea are still imprecise, and are estimated from global modelling studies to range from 0.5 to 23 million tons of plastic annually [[4], [5], [6], [7]]. Hence, a growing number of studies have focused on river plastic transport processes in recent years [[8], [9], [10]]. Available data suggests that the presence of river infrastructure, environmental factors (i.e. hydrological processes, channel morphology and riparian vegetation), and the characteristics of litter items (size, density, shape and surface tension) affect how plastic debris are transported, stored and remobilized in the river [8,[10], [11], [12], [13], [14]]. In addition, recent research suggested, that rivers may function as (temporary) reservoirs for land-based plastic pollution, as well as a potential source for its future remobilization [9,10,12,[15], [16], [17]].

The estuaries represent a transitional environment between continental and marine areas, but only limited studies were conducted to know how these complex systems contribute to plastic pollution dynamics at this interface [15,16,[18], [19], [20]]. Most of these studies focused on transfer and accumulation dynamics of (macro)plastics in estuaries. They highlighted the influence of tides and freshwater discharge, wind and interaction with the topography and morphology of riverbanks on plastic transport dynamic. For instance, [21] simulated plastic transport in the Chesapeake estuary (USA) and found that only 5 % of the annual microplastic transport was exported into coastal waters, whereas the overwhelming majority (94 %) beached on the estuarine shores. Some studies pointed out that estuaries are reservoirs for plastic pollution, where plastic accumulates over a long period of time before ultimately entering the sea [15,22]. Others studies highlighted the regular back-and-forth movement of waste within estuarine systems due to the tidal action, making it difficult to estimate the volume of plastic waste reaching the sea [8,16]. Understanding the processes of plastic deposition and remobilization along estuarine shores is fundamental for monitoring and modelling the fate of plastic pollution in these environments. The process of remobilization of plastic waste is particularly overlooked in the literature and still poorly understood. Surface-stored plastic waste may be remobilized from the estuarine shores by complex interactions between tides, freshwater discharge, vegetation, local morphology, and hydro-meteorological factors.

Therefore, this study aimed to address these gaps by investigating environmental parameters to better understand the processes affecting plastic debris dynamics in estuarine environment. Here, an *in situ* experimental study was conducted to investigate the mobilization and deposition of plastic debris (macro, meso and microplastics with size ≥3 mm) from an estuary bank into the water on a daily timescale during six complete tidal cycles. This investigation was conducted in a small estuary, the Slack estuary (located in Northern France) characterized by a semi-diurnal tide. The experimental design aimed to explore the complexity of the mobilization and deposition of plastic debris with regard to i) hydro-meteorological factors, ii) types of substrates present on the estuarine shores and, iii) type of plastics (macro, meso and microplastics), their shapes, sizes and polymer composition.

## 2 Materials and method

### 2.1 Study area and experimental design

**Fig. 1:**
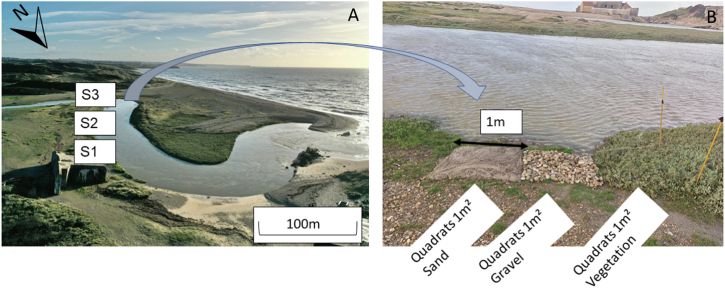
A) Location of the three selected sites (S1, S2, S3) along the bank of the Slack estuary. (B) The structure of each site consisting of three different substrates (vegetation, gravels and sand), a quadrat of 1 m^2^ each.

The Slack estuary is a small French coastal river located in northern France (coordinates 50°48′21.1"N 1°36′12.8"E). The Slack is a 21.8 km long river with a catchment area of 156 km^2^ and flows through several small municipalities before discharging into the English Channel (Fig. 1). The Slack estuary is characterized by a pluvial oceanic hydrological regime, maintaining a mean annual flow of 2.03 m^3^/s (http://www.hydro.eaufrance.fr), with relatively low fluctuations. High water flows occur from October to March with a mean flow exceeding 5 m^3^/s, while low water endures from April to September with a mean flow dipping below 1 m^3^/s. Depending on tidal coefficients, the water level and the river width changes from 1.5 to 6 m depth and 4–10 m width. The land surrounding this catchment area is used primarily for agricultural purposes, occupying 43 % of cultivated land, with further 20 % designated as built-up land, 20 % as grassland and woodland, and the remaining 17 % is beach land. Consequently, the estuary can be considered a low anthropized estuary, as it includes protected zones and remains a free-flowing unmanaged estuary Moreover, the estuary is accessible, and its banks are well representative of different types of substrates (vegetation, sand, and gravel).

To develop this methodology, first, a series of site inspections through several field campaigns were carried out on 9, 16, 17 and May 18, 2022 during both neap tides (tidal coefficient between 20 and 30) and spring tides (tidal coefficient 90–100). During the inspection period, a variety of weather conditions were observed, including precipitation, wind, and sun. The aim was to explore the spatial and temporal distribution of plastics and the types of plastic deposited on the estuarine banks under different tidal regimes. During these investigations, parameters such as bank altitude, river water level and types of substrates were carefully observed and recorded. In addition, clean-up and collection of plastics along riverbanks was carried out during this work observation, in order to precisely identify the type of plastic found and reuse the collected plastics for mobilization. This fieldwork served as a crucial decision-making tool, and helped in guiding the research methodology. This allowed to choose the spring tide period for the experiments because all the quadrats were covered by water during the high tide, enabling to study the impact of the tide on mobilization and new plastics deposited by the tide. It also enabled to identify appropriate substrates and determine the types, sizes and polymer compositions of plastics to focus on. The River-OSPAR method [22] guided the selection of the 1 m^2^ test areas and the categorization of the plastics collected. It was complied with the first criterion, by standardizing the sample area to 1 m^2^, and the second criterion, by classifying the plastics by size, type, material composition and source. The third criterion, which considers site characteristics such as the shape of the bank, vegetation and proximity to sources of pollution, was adjusted by selecting a segment of the estuarine bank parallel to the water, straight and without meanders. This adaptation simplified the analysis of plastic accumulation by minimizing the complex influences of water flow around curves.

The experimental design consisted of three sites (S1, S2 and S3 – Fig. 1A) with a distance of 8 m between each one located along the same estuarine bank to ensure consistency and similar environmental conditions. Each site was composed of three quadrats of 1 m^2^ representing the three distinct substrates present in this estuary: vegetation, gravel and sand (Fig. 1B). The upper edge of each quadrat was positioned at the high-water line, which corresponds to the upper limit of the water level when the tide rises during spring tides. In this way, each quadrat was always submerged during flood tide. Vegetation consisted of sea purslane (*Halimione portulacoides*) of about 20 cm high. Gravel quadrats were characterized by a height of 8 cm, constructed with typical medium to large river rocks (1–5 cm) commonly found along the estuary. The sand quadrats consisted of a 10 cm thick layer of sand carefully sieved using a 500 μm and 2 mm sieve before use. When constructing the sand quadrats, the slightly wet sand was sieved to ensure that it was free of any microplastic contamination within the studied size class. Prior to the installation of quadrats, the sites (S1, S2 and S3) were systematically cleaned from all kind of debris including vegetation (leaves, branches, algae, etc.) and plastic, to ensure a pristine environment and to establish a baseline. To set up the experiment, first a segment parallel to the waterline was identified, where existing vegetation was submerged by flood tide. Three areas were selected and delimited the vegetation within each area. Adjacent to each naturally existing vegetation patch, two quadrats were installed, one filled with gravel and the other with sand. Both sand and gravel were collected from the same estuary (few meters away, refer to Fig. 1) as the study segment, which naturally did not favor the accumulation of these materials at higher water levels. Before depositing these two new substrates next to the vegetation quadrat, all the elements present in these selected sites were removed (vegetation and plastics).

This installation of gravel and sand took place during the first survey. The team arrived on site early (4 h before flood tide) to perform the installation. In subsequent surveys, the team continued to arrive early to check for any site damage, and made any necessary repairs if the gravel or sand had eroded. A team of 4 people had sufficient time to set up the quadrats efficiently. Two members concentrated on preparing the layout of the 1 m^2^ quadrats. Then the four members began to carefully clean each quadrat of any plastic or tidal debris. To collect material, one member collected gravel (about a minute's walk from the quadrats), while another collected and sieved sand. A stainless-steel sieve was used, first with a 2 mm sieve and then with a finer 500 μm sieve, to ensure consistency. The sieved sand was placed in a metal bucket for transport. The other two people were responsible for installing each quadrat at the correct height. Naturally, there were switching roles between the members. Arriving early in the morning, the team used personal protective equipment (e.g. life jackets) and lighting to complete the quadrats safely and on schedule.

**Fig. 2:**
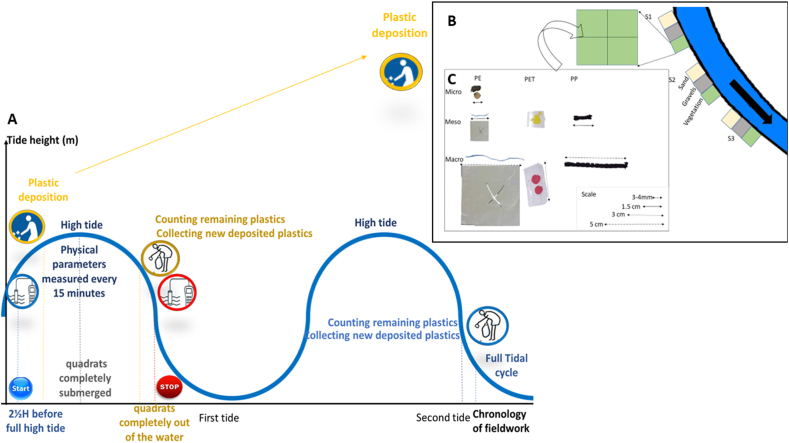
(A) Steps of the adopted methodology during this study. (B) Detailed methodology description: site triplicates and substrate quadrat (1 m^2^) subdivision schematic. Sites (S1, S2, and S3) and substrates (Green for Vegetation, Gray for Gravel, and Yellow for Sand). Each quadrat was divided into four sub-quadrats (0.5 m × 0.5 m). Ten plastics were placed in each sub-quadrat resulting in 40 plastics per quadrat. The arrow represents the flow of the river. (C) Different plastics collected and deposited on cleaned quadrats over the estuarine bank. Type of used plastics (films (PE), fragments (PET), cords (PP), fibers (PE) and pellets (PE)), and size of used plastics (macro, meso and micro).

The plastic set, made from plastic waste previously collected during the inspection observation, originating mostly from marine activities such as cords and fibers, was manually and carefully placed on each cleaned quadrat. Each set consisted of the same 10 pieces of plastic debris, classified into three size classes: micro (3–4 mm), meso (5–25 mm), and macro (>25 mm). Specifically, this included two pellets made of polyethylene (PE) ranging from 3 to 4 mm, four mesoplastics of around 15 mm, and four macroplastics between 30 and 50 mm. For microplastics, only pellets were considered, given that they were found frequently along the banks. Meso and macro sized categories included four types of shapes: films, fibers, fragments, and cords. These plastics were made from three types of polymers: polyethylene (PE), polyethylene terephthalate (PET), and polypropylene (PP). The set included a PE film, a PET fragment, a PE fiber, and a PP rope for each macro and meso category, while the micro category was represented by two PE pellets, as shown in Fig. 2 (C).

Each quadrat was divided into 4 sub-quadrats (50 × 50 cm) and a plastic set was deposited into each sub-quadrat, totaling 40 plastics per quadrat (Fig. 2B). Overall, during each survey, a total of 360 pieces of plastic debris, ranging in size from 3 to 50 mm, were deposited in the 9 quadrats divided into 3 identical triplicates (Fig. 2B). To differentiate the plastics belonging to each quadrat, they were specifically marked with a waterproof color. Nine colors were used (light green, dark green, white, pink, yellow, green, light blue, dark blue, and red), each corresponding to a single quadrat. Thereby, it was possible to study the flux of plastics specifically in relation to the substrate on which they were manually deposited (Fig. 2).

### 2.2 Experimental procedure

Six field surveys were conducted between June 2022 and July 2023 (Supplementary data) in order to explore the deposition and mobilization of plastics from the banks to estuarine waters after a full tidal cycle during spring tide (tidal coefficients >70). For the experimental strategy, survey dates were chosen in order to allow observations during the daytime and correspond with tidal coefficients that allowed the quadrats to be completely covered and uncovered during the tidal cycle. Each survey spanned 24 h, divided into two stages, each corresponding to a semi-diurnal tidal cycle (12h).

Sub-surface hydrological parameters, including water turbidity (measured with a HANNA Iso turbidimeter, HI98713), salinity and temperature (measured with a HANNA multiparameter, HI 9828) and water speed (measured with a Global Water Speed Probe current meter, brand Xylem), were measured 10 cm below the water surface, in the middle of the estuary, parallel to the sites, directly in front of the middle site. During the surveys, the depth of the water made it possible to take measurements directly from the riverbanks. Team members wore waterproof waders and followed all safety protocols. Wind speed and velocity were also measured at 1 m above the sites using an Extech CFM/CMM AN100 thermo-anemometer. This data collection started two and a half hours prior to high tide and stopped when the water line returned to its initial state, following the first low tide, only. For the second low tide, environmental parameters were measured only two and a half hours after the high tide. (Fig. 2A). The surveys days were selected according to the occurrence of low tides, ensuring that the water level was low enough to expose the quadrats during the day. During these periods, mobilization of manually placed plastics was closely monitored after each high tide when the quadrats were totally uncovered and the new deposited plastics were collected after each observation (Fig. 2A), allowing a study of mobilization and deposition over a complete tidal cycle. According to the OSPAR methodology, observations were conducted on the surface of the banks. A team of 4 people observed each 1 m^2^ quadrat, in order to thoroughly examine the plastics and to minimize the risk of underestimation. Gravel was inspected both on the surface and between the gaps, without disturbing the material. The same approach was applied to sand, with all particles on the surface or partially buried being observed without digging into the sand. During most of the field surveys, the sand naturally formed wave-like shapes. Observations of the deposits were made by the same team and using the same techniques as for mobilization (at the surface, without digging). Despite the care required for this type of observation, especially for the 3 mm pellets (lower size limit), some were spotted in the vegetation.

#### 2.2.1 Polymer identification

Micro and Macro Raman spectroscopy were used to identify the types of polymers of the plastics used (manually deposited) and seen in this study (deposited by tide). Micro Raman spectroscopy (XploRA™ PLUS, Horiba), a technique that uses laser excitation to measure the vibrational spectra of microscopic samples, was carried out using two laser wavelengths: 532 nm and 785 nm. The focal lengths of Micro Raman spectrometers generally range from 200 mm to 800 mm, or even more, depending on instrument design and application. Raman spectrometer gratings range from 300 gr/mm (low resolution) to 1800 gr/mm (high resolution), with specialized gratings such as 2400 gr/mm available for more precise applications. For Macro Raman (MacroRAM™, Horiba) analysis, a laser wavelength of 785 nm was used, with laser power adjustable between 7 mW and 450 mW. The Macro Raman spectrometer had a focal length of 115 mm and a spectral range from 100 cm^−1^ to 3400 cm^−1^. Spectroscopic analysis was carried out using LabSpec 6 software, which offers comprehensive tools for advanced Raman analysis. Micro-Raman spectroscopy was particularly useful for identifying thin or small plastic such as pellets, fibers or other shapes.

### 2.3 Statistical analysis

As data did not meet the parametric hypotheses of normality (Shapiro-Wilk test), non-parametric statistical tests for comparison were used. The percentage of mobilized plastics was tested for spatio-temporal variations using the Chi-squared test and the PERMANOVA “Bray-Curtis dissimilarity” permutation test (N = 9999), followed by a post-hoc test. The abundance of deposited plastics was tested using the Mann-Whitney test and Kruskal-Wallis test. Dunn's test was used for post-hoc comparisons. Variations in plastic mobilization condition were analyzed using a Canonical Correlation Analysis (CCA) performed as a constrained ordination technique to explore the influence of different factors namely plastic size, characteristics and hydro-morpho-sedimentary factors. Before analysis, data were standardized using the Hellinger distance transformation, followed by centering and reduction. The statistical software R Studio was used for all statistical analysis (R Core Team, 2022). Specifically, the *Stats* package of R software was used for the Shapiro- Wilk test, Kruskal-Wallis, Man-Whitney test, Chi squared test, adonis2 package was used to perform PERMANOVA test, while the vegan packages of R were used for the CCA and variation partitioning. The level of significance was set at α = 0.05.

## 3 Results

### 3.1 Plastic mobilization

#### 3.1.1 Variation of plastic mobilization according to tidal cycles, time, plastic size and morphology

**Fig. 3:**
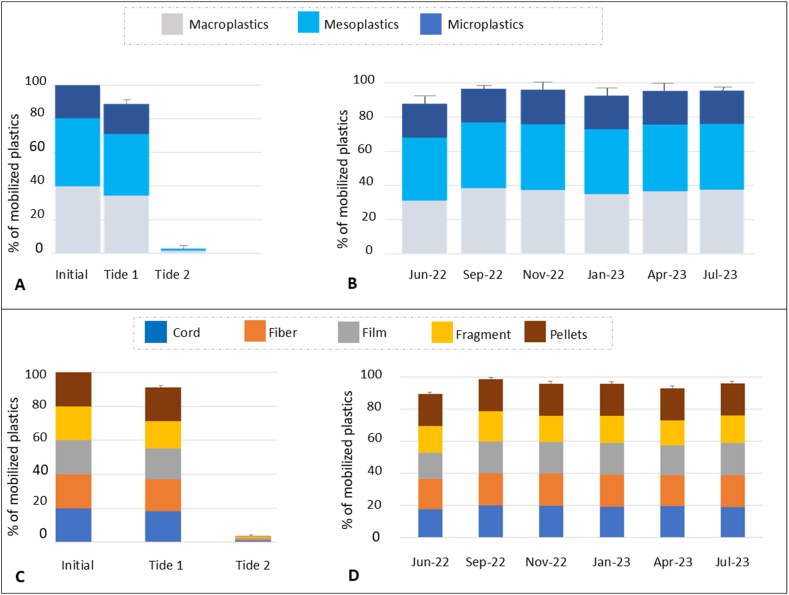
(A) Initial percentage of placed plastics and percentage of mobilized plastics (mean + SD) per square meter during the first and second tides according to their size category (macro, meso and micro) and considering all surveys together; (B) Percentage of mobilized plastics classified by size during each survey after a complete tidal cycle; (C) Percentage of mobilized plastic according to their shape after a complete tidal cycle, considering all surveys; and (D) Percentage of mobilized plastics according to their shape during each survey.

During the six experimental surveys conducted between June 2022 and July 2023, an average of 94.48 ± 3.1 % of the plastics deposited in each quadrat were mobilized after a full tidal cycle (Fig. 3A). The majority of plastics (91.01 ± 4.15 %) were mobilized after the first tide with only 3.7 ± 2.8 % plastics mobilized after the second one. After a full tidal cycle, the percentage of plastic mobilized ranged between 89.44 ± 3.15 % on June 16, 2022 and 98.61 ± 0.48 % on September 12, 2022 (Fig. 3B). There was no significant temporal variation in the mobilization rate among the six campaigns (Chi-squared test, p-value >0.05). During these experiments, mesoplastics exhibited a higher mobilization rate over a complete tidal cycle (96.29 ± 2.47 %) compared to macroplastics (90.62 ± 7.26 %) while microplastics were completely mobilized into the water (100 % pellets). With regards to plastic size, there was no significant variation in the mobilization rate among the six surveys (Chi-squared test >0.05) (Fig. 3B).

Five types of plastics were used in this study: cords, fibers, films, fragments, and pellets. Considering all surveys together, fibers and pellets tended to exhibit the highest mobilization rate, with 100 % of manually deposited pellets being mobilized after a complete tidal cycle followed by fibers (98.61 ± 2.32 %), cords (95.6 ± 4.4 %), films (94.9 ± 7) and fragments (84.72 ± 5.55 %) (Fig. 3C). Over all the surveys, there was no significant variation in the mobilization rate of each shape of plastic (cord, fiber, film, fragment) (Fig. 3D) (Chi-squared test >0.05) and the distribution of mobilized plastic shapes appeared uniform, with no notable differences observed (PERMANOVA test, F = 1.53, p-value = 0.16).

#### 3.1.2 Influence of substrate type on plastic mobilization

**Fig. 4:**
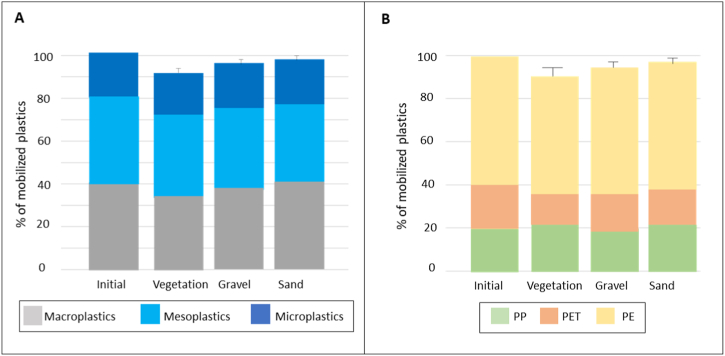
Initial percentage of placed plastics and percentage of mobilized plastics (mean + SD) across the three substrates (Vegetation, Gravel and Sand) according to (A) Size of plastic, and (B) Type of polymer.

Three types of substrates were used in this study (vegetation, gravel and sand). Overall, the mobilization rate of plastics showed no significant differences for the various substrates (Chi-squared, p-value >0.05) (Fig. 4 A). The lowest mobilization rate was recorded for vegetation (90.6 ± 2.5 %), followed by gravel (95.56 ± 1.4 %), and sand (97.55 ± 1.19 %). Overall, the plastic size classes (micro, meso and macro) showed no significant variation in mobilization rates based on the type of substrate, regardless of plastic shape (PERMANOVA, F = 3.6, p = 0.15). Furthermore, taking mesoplastic shapes into account, they did not reveal any significant difference in their mobilization between substrates (PERMANOVA, F_meso_ = 2.5 p_meso_ = 0.08). However, with regard to plastic size, macroplastics exhibited significant different trends of mobilization rates, expressed as a percentage of the total remobilized plastics, between sand and the two other substrates (PERMANOVA, F = 4.05, p = 0.03, post-hoc <0.05), with a lower mobilization rate on vegetation (33.19 ± 6.33 %) and gravel (36.38 ± 2.02 %) compared to sand (38.9 ± 1.4 %). Among these macroplastics, macro fragments exhibited the lowest mobilization rate with an average of 61.1 ± 21.51 % of the placed macro fragments remobilized from vegetation and 75 ± 7.45 % from gravel, while macro fibers showed the highest rates of mobilization of the placed macro fibers remobilized from vegetation to water, with an average of 98.61 ± 3.4 %. With regard to the sand substrate, plastic types of all sizes showed similar mobilization rates, ranging from 94.4 % ± 4.3 for macro fragments to 100 % for macro cords and fibers. In addition, on the gravel, 100 % of the meso cords were mobilized in the water. The colors were used to identify potential movements of plastic between quadrats, but no significant migration was observed, suggesting that plastics were occasionally mobilized in the water or migrated to another point outside the study area. It is important to note that the plastics observed were exclusively on the surface of the gravel and sand substrates, as no digging or substrate disturbance was carried out. For substrates such as gravel and sand, it is indeed possible that microplastic pellets have become lodged between the gravel particles or have been buried in the sand, which could lead to being overlooked.

The mobilization rates of the three polymers studied (PP, PE and PET) showed a similar distribution pattern between the three substrates (Fig. 4B). Polyethylene (PE) was present in different shapes (pellets, films and fibers) and sizes (micro, meso and macroplastics). A consistent mobilization rate of PE was observed for all shapes and sizes, ranging from 90.2 ± 1.7 % to 98.6 ± 3.4 %.

#### 3.1.3 Effect of hydro-meteorological factors on plastic mobilization

**Fig. 5:**
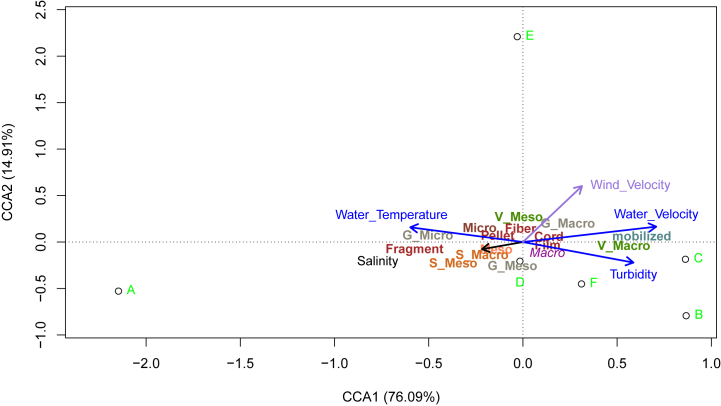
Canonical correlation analysis (CCA) analyses of environmental parameters (Salinity, Turbidity (NTU), Wind velocity (m/s), Water velocity (m/s) and Water temperature (°C)), mobilized plastics size (Macro, Meso and Micro) and form (Film, Fragment, Fiber, Cord and Pellet) of plastic mobilized from each substrate (Vegetation (V), Gravel (G) and Sand(S)) in the Slack estuary. White circles with the green letters represent the six conducted surveys (A = June 6, 2022; B = September 12, 2022; C = November 24, 2022; D = January 24, 2023; E = April 20, 2023; F = July 6, 2023). The blue axes refer to environmental parameters related to water, while the purple axes represent wind. The black axis indicates salinity. Green labels marked with the letter 'V' correspond to vegetation, gray labels with 'G' relate to gravel, and orange labels with 'S' are associated with sand. The red wine color is used for the shape of plastics (e.g., fragment, cord, film, pellet, fiber).

With regard to hydro-meteorological factors, salinity seemed to have the least impact on plastic mobilization, unlike turbidity, water velocity, water temperature and wind velocity (Fig. 5). In addition, plastic mobilization tended to be lower when the wind direction was oriented towards the banks of the estuary. In June 2022 and April 2023, the mobilization rates were lower, with averages of 84.2 ± 2.6 % and 93.05 %, respectively, of mobilized plastics/m^2^. In comparison, the rates during other periods ranged from 96.1 ± 1.3 % to 98.6 ± 1.7 % of plastics/m^2^. There was a positive correlation between water velocity and global mobilization rate. Macroplastic mobilization was positively influenced by higher levels of turbidity, water and wind velocity. Mesoplastics mobilization were less affected by these environmental parameters but seemed to be linked to water temperature and salinity.

Regarding plastic shapes, film and cord mobilization were more influenced by turbidity levels, water and wind velocities, while fragment mobilization showed a stronger correlation with salinity. Fiber mobilizations exhibited similar responses to water temperature. The influence of environmental parameters on the plastic mobilization also depended on the substrate. The mobilization of plastics (mainly macro) from gravel and vegetation showed a positive relation with wind and water velocity. While, macroplastics on sandy substrates showed a distinct trend, where their mobilization was mainly affected by variations in salinity.

### 3.2 New deposited plastic

**Fig. 6:**
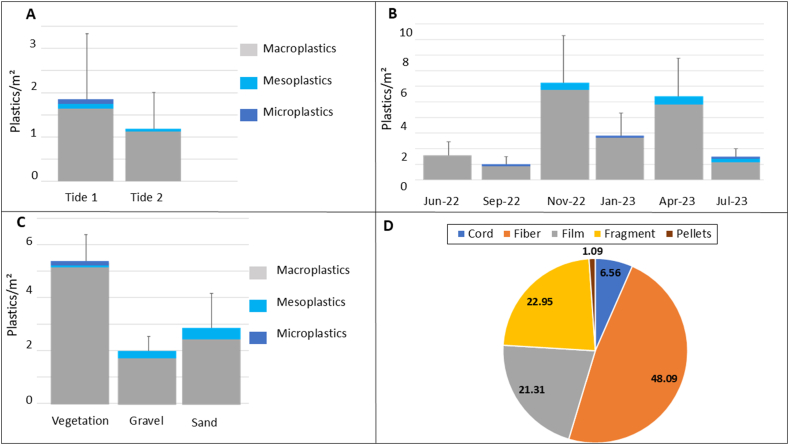
A) Abundance (mean ± SD) of deposited plastics (plastics/m^2^) during first and second tide considering the size category (macro, meso and micro) and all surveys together; (B) Abundance (plastics/m^2^) and size distribution (macro, meso and micro) of deposited plastics after a full tidal cycle considering each survey alone, (C) Abundance of deposited plastics (plastics/m^2^) on each substrate (Vegetation, Gravel and Sand) considering the size category and (D) Percentage of deposited plastics according to their shapes considering all surveys after a full tidal cycle.

After the first tide, an average of 1.84 ± 1.4 plastics/m^2^ was deposited, without significant difference compared to a mean of 1.15 ± 0.8 plastics/m^2^ after the second tide (Fig. 6A) (Mann-Whitney test, p-value >0.05). However, significant differences in the sizes of the deposited plastics were observed (Kruskal-Wallis test, p-value <0.05). More specifically, macroplastics were the most deposited plastics during the first tide, with an average of 1.7 ± 1.3 plastics/m^2^, compared to mesoplastics (0.07 ± 0.1 plastics/m^2^) and microplastics (0.03 ± 0.05 plastics/m^2^). Microplastics were deposited exclusively during the first tide (Fig. 6A). At the second tide, macroplastics were still the most frequently deposited plastics, with an average of 1 ± 0.6 plastics/m^2^ (Kruskal-Wallis test, p-value <0.05) (Fig. 6A).

Considering each survey, significant fluctuations in the abundance of plastics deposited over time were observed (Kruskal Wallis test, p-value <0.05). The lowest abundance was observed on September 12, 2022 (0.99 ± 0.33 plastics/m^2^), while the highest abundances were recorded on November 24, 2022 and April 20, 2023, with 6.1 ± 3.1 and 5.35 ± 2.45 plastics/m^2^ respectively (Dunn's test, p-value <0.05) (Fig. 6B). Macroplastics were deposited during each survey whereas meso and micro plastics were deposited during only certain surveys.

Regarding the type of substrate, vegetation trapped more plastics than the other types of substrates with an average of 5.37 ± 1.01 plastics/m^2^ after a full tidal cycle compared to 1.94 ± 0.59 plastics/m^2^ deposited on gravel and 2.83 ± 1.32 plastics/m^2^ deposited on sand (Fig. 6C) (Kruskal-Wallis, p-value <0.05). Furthermore, vegetation accumulated more macroplastic, with an average of 5.11 ± 2.6 macroplastics/m^2^, compared to gravel and sand, with an average of 1.8 ± 1.6 and 2.33 ± 3.1 macroplastics/m^2^, respectively (Kruskal-Wallis, p-value <0.05). While macro and mesoplastics can be found in all substrates, microplastics were deposited exclusively on vegetation with an average of 0.165 ± 0.18 microplastics/m^2^. Mesoplastics were more present on sand and gravel than on vegetation. Indeed, sand had an average of 0.5 ± 0.8 mesoplastics/m^2^, and gravel had 0.11 ± 0.17 mesoplastics/m^2^, compared to vegetation which had a lower average of 0.095 ± 0.2 mesoplastics/m^2^ (Kruskal-Wallis, p-value <0.05). With regard to the shapes of the deposited plastics and considering all campaigns, fibers were the most predominant, accounting for 48.09 % of the total deposited plastics followed by fragments (22.95 %), films (21.31 %), cords (6.56 %) and, pellets (1.09 %) (Fig. 6 D).

Five types of polymer were identified among these deposited plastics: polypropylene (PP), polyethylene (PE), polystyrene (PS), polyethylene terephthalate (PET) and polyvinyl butyral (PVB). PP and PE were the predominant polymers, collectively accounting for more than 70 % of the deposited plastics.

**Fig. 7:**
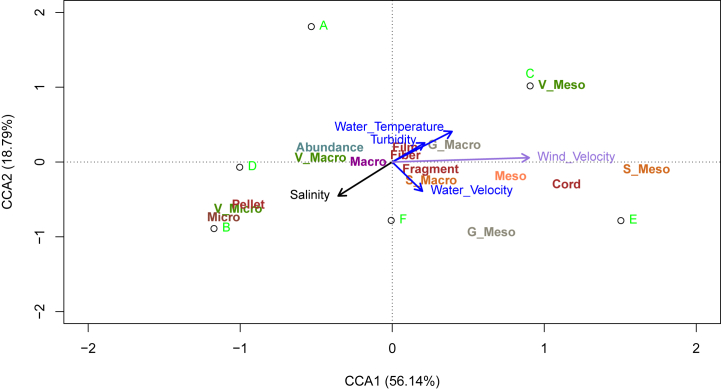
Canonical correlation analysis (CCA) analyses of environmental parameters (Salinity, Turbidity (NTU), Wind velocity, Water velocity and Water temperature), Deposited plastics: Abundance, size (Macro and Meso) and form (Film, Fragment, Fiber and Cord) from each substrate (Vegetation (V), Gravel (G) and Sand(S)) in the Slack estuary. White circles with the green letters represent the six conducted surveys (A = June 6, 2022; B = September 12, 2022, C = November 24, 2022, D = January 24, 2023, E = April 20, 2023, F = July 6, 2023). The blue axes refer to environmental parameters related to water, while the purple axes represent wind. The black axis indicates salinity. Green labels marked with the letter 'V' correspond to vegetation, gray labels with 'G' relate to gravel, and orange labels with 'S' are associated with sand. The red wine color is used for the shape of plastics (e.g., fragment, cord, film, pellet, fiber).

Regarding the impact of environmental parameters on the deposition of plastics, the two surveys with the highest abundance of plastics deposited (C and E in November and April respectively) were positively correlated with wind velocity (Fig. 7). This parameter seems to positively influence the deposition of macro and meso-sized plastics, particularly those with a cord-like shape. Salinity seems to favor the deposition of pellets.

## 4 Discussion

The fate of plastics in river and estuarine systems is strongly dependent on three processes, namely (1) the transport, (2) the deposition and accumulation, and (3) the mobilization. Understanding these processes along river and estuarine shores is fundamental for monitoring and modelling the fate of plastic pollution in these environments. In the present study, the mobilization and deposition of plastics along estuarine banks was assessed on a daily basis during six complete tidal cycles.

### 4.1 Plastic mobilization

During the six experimental surveys, most plastics (around 94 %) were mobilized after a daily tidal cycle with the majority being mobilized after the first tide. These observations align with previous studies indicating that in tidal rivers and estuaries, tidal dynamics influence the transport and export of plastic items into the ocean [10,16,23]. Hydrodynamics in tidal rivers and estuaries are influenced mainly by tides and freshwater discharge. In tidal rivers, it has been shown that plastic transport rates vary greatly over time and are positively correlated with river discharge [24]. High river discharge periods, potentially due to seasonal variations, as well as the amount of rainfall, can impact the transport of plastics [14,[25], [26], [27], [28]]. In the present study, no significant variation in plastic mobilization was observed between the six field surveys carried out over the different seasons. The pluvio-oceanic climatic characteristics of the studied site, with regular rainfall over the year combined with low river flows (between 2 and 5 m^3^/s), may have led to the absence of temporal variations in plastic mobilization. Results indicate that in a small macrotidal estuary, tidal cycle (flood and ebb) play a significant role in plastic mobilization of surface-deposited plastic items on estuarine banks. However, the physical characteristics of plastics, some hydrometeorological factors and the type of estuarine bank substrate may influence the mobilization probabilities. This is in line with some studies that suggest that debris retention/mobilization varies much more with habitats (e.g. riverbank morphology, vegetation density, hydrodynamic conditions) and item behaviours (e.g. shape, size, density) than with seasons [10,29,30], with the exception of extreme events like storms or highest river discharges [9,24].

Certain shapes of plastics, namely fibers showed a high mobilization rate during the first tide highlighting the distinct behavior of plastics in response to tidal movements. The shape, size, and polymer composition of plastic waste have already been identified as the main factors influencing plastic transport [11]. Furthermore, macroplastic fragments tended to show lower mobility than other shape of plastics, which is consistent with other studies [9,26]. Pellets were all mobilized into the water after a complete tidal cycle. Indeed, smaller particles are observed to float further on the surface of the water as the water level increases and can be mobilized more easily [8,31].

Polymer types also influenced plastic mobilization in the present study. The lowest mobilization rate was observed for PET, attributed to its higher density 1.38 g/cm³, and making it less buoyant compared to other studied plastics [29]. In contrast, the lighter particles deposited, such as films (PE), fibers (PE) and pellets (PE), were often mobilized after a semi-diurnal tidal cycle. Indeed, PE, a polymer with low density (0.91 g/cm³), might exhibit higher mobilization rate. This can be attributed to significant salinity fluctuations at the estuarine mouth, as noted by Li et al. (2021) which may modify the buoyancy of low-density plastics and promote their mobilization. Plastic transport can be determined by the balance between buoyancy, surface tension and turbulence in the medium [13].

Observations indicated that some hydrometeorological factors may amplify the mobilization of plastics from the estuarine banks into estuarine waters. Wind and water velocity, turbidity, and water temperature were the factors that influenced the most the plastic mobilization. There was a positive correlation between water velocity and global plastic mobilization rate, highlighting the role of water velocity, associated with the tidal dynamic, as drivers of plastic mobilization and transport in estuarine systems. The mobilization of the polyethylene fibers was influenced by changes in water temperature. In this study, a slight increase in water temperature resulted in the transport of lighter plastics, probably due to changes in water density or currents. This increase in water temperature also indicates the rise of the tide in the study area. Moreover, as measurements were taken in different seasons, temperature variations reflected seasonal differences, but no clear trends were observed between seasonality and PET fiber mobilization. Macroplastic mobilization was positively influenced by higher levels of turbidity, water and wind velocity. For instance, the wind affected plastic mobilization through its direction and velocity. Wind speed seemed to have a greater influence on macroplastics mobilization. Higher levels of mobilized plastics tend to be observed when the wind blew towards the water (WSW and SE directions). A study by [33] conducted on the shores of the Tamar Estuary in the United Kingdom revealed that leeward habitats can serve as debris sinks. This role of wind in plastics transport was also confirmed by Schwarz et al. (2019). Water velocity plays a crucial role in the transport of plastics along river banks and in coastal and estuarine environments. The increase in water velocity can lead to higher transport rates of influx of plastics [34]. In estuaries, tidal dynamics further complicate these patterns as they can reverse surface flow directions, affecting both the retention and net transport of plastics in tidal rivers and estuaries. Although the study site is located at the estuarine mouth where high variation in salinity can be observed during the tidal cycles (from 0.9 to 32 PSU), salinity was the parameter with the least influence on plastic mobilization. However, previous studies showed that the salinity gradient which influences density and therefore buoyancy, can also induce the movement of particles into water [35,36].

Furthermore, plastic mobilization was also influenced by the nature of the substrates. Vegetation showed the lowest plastic mobilization rate, which is consistent with other studies highlighting that the vegetation tends to trap more plastics and is considered as a sink for plastics [12,26,37]. For instance, riparian vegetation along riverbanks in estuaries can act as both a support and a reservoir for plastics [17]. At the study site, sea purslane (*Halimione portulacoides*), a shrubby vegetation type, was predominant along the estuarine banks. It was noted that shrubby vegetation, along with arboreal plants, tend to capture more macroplastics compared to herbaceous, reed, and bush varieties [37]. The complex structure of the substrate, characterized by dense vegetation on site, made it challenging for plastics to disperse into the water. On the other hand, the rate of plastic mobilization was the highest on sand, followed by gravel substrate. This is consistent with the findings of [38] who observed higher plastic accumulation in the vegetation compared to the sandbanks. These differences in plastic mobilization rates could be linked to the different interaction of these substrates with water. Indeed, water can impact the plastic movement patterns: saline water infiltrates gravel gaps resulting in plastic transport, while physical properties of sand change with water interaction, providing a smooth surface for plastics and facilitating particles movement [39]. Results suggested that particles characterized by a large cross-sectional area, low density and flat shape had difficulty entering the substrates.

### 4.2 Plastic deposition

New plastic deposits by tide were also observed on the different substrates during the six tidal cycles studied. The alternation of submersion and exposure of estuarine banks due to tides enhances the accumulation of natural and human-made buoyant debris in estuaries [15,23,40]. An average of 3.33 ± 1.8 plastics/m^2^ was deposited during a complete tidal cycle. There is limited data on the daily accumulation of plastics in estuaries. Observations indicate a higher density of plastic accumulation compared to the results observed on sixteen riverbanks along the Dutch Meuse river where daily macroplastic accumulation ranged from 0.25 to 0.01 items/m [29]. In the Warnow estuary (Baltic Sea), density of large micro and mesoplastic accumulation varied from 0.3 to 31 particles/m^2^ [41]. Data are also similar to those observed along beaches worldwide. On five Cape Town beaches, daily marine plastic debris accumulation rates were 0.36–2.96 items/m^2^ [42].

Macroplastics were the most deposited plastics during the tidal cycle. The predominant plastic deposits were fibers, commonly used for fishing and marine activities. It was shown that tides and waves can facilitate the input and accumulation of marine waste in estuarine areas [43]. Other studies highlighted that the accumulation of plastic in estuaries is mainly influenced by land-based waste [19,20,32,44,45]. The observation of marine origin plastic deposits in the present study, contrary to most studies, is more likely due to the lower level of human impact of the Slack estuary compared to other studied estuaries. Moreover, its location at the English Channel, a major thoroughfare for Northern European fishing boats, contributes to the marine origin of the observed plastic debris.

Most of the deposit plastics were observed in the vegetation substrate, which highlighted the role of estuarine bank vegetation as a natural trap for plastics transported by tides. Vegetation tended to trap 2 to 3 times more plastics than the other types of substrates (gravel and sand). This observation is in line with previous research showing the significance of riparian zone in the accumulation and capture of plastics [32,37]. Microplastics were exclusively deposited in vegetation, consistent with the observations of Han et al. (2022) indicating that increased plant density contributes to the interception of microplastics. In contrast to mobilization, a significant temporal variation in deposited plastic was observed. The role of environmental parameters highlighted the deposition of these new plastics during the various surveys. Specifically, the wind velocity directly promoted the deposition of plastics on the estuarine banks in November and April. This wind seems to facilitate the transport of mesoplastics, mainly in the form of cords, while the deposition of macroplastics is more closely related to salinity. These points demonstrate the importance of considering all environmental factors when studying plastic pollution at sites.

The role of estuaries on plastic transfer between rivers and the ocean, and net retention on estuarine banks, remain largely unresolved. A particle dispersal model in the Bay of Brest (France) showed that about 60 % of MPs were flushed out of the estuary after being exposed to the daily tides for ten days [46]. Within tidal cycles, plastic flux close to the river mouth has been found to be the same during both ebb and flood tide, suggesting that the actual net transport from rivers into the sea is very limited [22]. Current findings made it possible to estimate the plastic flux during a tidal cycle and results suggest that on average less than 0.03 plastic/m^2^ remains on estuarine banks after one tidal cycle suggesting that the daily net retention is very limited. However, in vegetated substrate, the daily net retention may be higher with 0.06 plastic/m^2^ highlighting the potential for dense vegetation to serve as a potential retention area for plastic waste accumulation.

## 5 Limitations of the study and recommendations for future research

In this study the focus was on three categories of plastics (macro, meso and microplastics) to investigate the mobilization and deposition of plastic debris on an estuarine bank. As far as microplastics are concerned, the difficulty of detecting them during the observations in the field led to limit this study to pellets of 3–4 mm in size. Moreover, the number of microplastics (pellets) used in the experiments is low and not representative of all type of microplastics found in nature although at the study site, where many pellets were found along the banks. This choice limits conclusions in the current study concerning the mobilization and deposition of microplastics along estuarine banks. Furthermore, even though the plastic pellets remained detectable to the naked eye when examined closely, it was attempted in the present study to reduce observation errors by systematically having four people observing each 1 m^2^ area. However, the possibility of error still exists especially since pellets detection is based on surface observations without the use of digging methods and hence may limit the ability to detect buried plastic pellets or small-sized fibers. To avoid underestimating plastic mobilization or deposition on substrates, it is recommended to add surface sand sieving step (2–3 cm with a fine-mesh size) as well as gravel collection and rinsing into the sampling quadrats. These techniques would enable counting of any plastics that may have become buried under the sand or squeezed between the gravel. This could improve the detection accuracy of small plastics and enable a more in-depth assessment of plastic pollution in different types of substrate. Moreover, for future studies, it's suggested to use color fluorescence or fluorescent microplastics combined with the use of ultraviolet light torch to better track and recover plastics and minimize errors.

Another limitation of this study is the absence of control samples consisting of quadrats not impacted by the water during the tidal cycle. The lack of control makes it impossible to ensure that the mobilization and deposition of plastics is due solely to the tidal cycle. For future work, it is recommended to include control samples in order to assess the effects of wind and other environmental factors, such as rain, on plastic mobilization and deposition over the same period following manual deployment of the plastic set. They should be placed away from the influence of tidal effects. This ensures that the area under investigation will not be disturbed by water level changes. The control samples may also be useful to assess the recovery experiments to ensure that the methodology in place is effective in recovering the different categories and size of plastics used.

Other methodological improvements could also provide a more robust data set, such as conducting experiments focusing each on a single size, shape or polymer, in order to better analyze the influence of these plastics' characteristics on their mobilization and deposition.

Conducting surveys at higher frequencies and at different seasons could provide a more complete understanding of plastic dynamics over time, allowing statistical analyses that reveal trends and clearer relationships between variables. Finally, the use of technologies to monitor and track the live transport of plastics could considerably improve the understanding of their movement and behavior, within a quadrat or along the estuary zone (e.g. GPS-trackers). However, these approaches can currently only be applied to plastics of a certain size (macroplastics) due to the size of the trackers.

## 6 Conclusions

This research presents a unique dataset describing the mobilization and deposition of plastics in a small macrotidal estuary at a daily basis over six tidal cycles. Results showed that tidal cycle plays a significant role in plastic mobilization and deposition on estuarine banks. The mobilization rate is less variable than the deposited plastics and seems to be affected in a more complex way by hydrometeorological factors. However, the effect of wind may be particularly important for the deposition of new plastics. Moreover, the nature of the substrate directly impacts the mobilization and deposition of plastics, highlighting the potential of dense vegetation that may serve as a limited retention area for plastic waste accumulation. Most plastics (around 94 %) were found to be mobilized after a complete tidal cycle while an average of 3.33 ± 1.8 plastics/m^2^ was deposited during the same period. This means that the proportion of plastics deposited by the tides accounted for 3 %–15 % of the plastics deposited manually. The majority of these deposits originated from marine activities, underlining the role of the environment surrounding the estuary in the type of plastic pollution present on estuarine banks. Current findings suggest that the daily net retention is very limited since most plastics are mobilized. The net transport, retention and the time scales of retention along estuarine banks remain largely unresolved. To better understand the role of estuaries on plastic transfer between rivers and the ocean, more studies are needed to the link between the mobilization capacity of plastics, the observed deposits and their potential sources, particularly in anthropized estuaries. These efforts are essential for designing targeted mitigation strategies and improving the ecological integrity of estuarine habitats.

## CRediT authorship contribution statement

**Rosa Sawan:** Writing – original draft, Visualization, Methodology, Investigation, Formal analysis, Data curation, Conceptualization. **Périne Doyen:** Writing – review & editing, Visualization, Supervision, Methodology, Conceptualization. **Guillaume Veillet:** Investigation. **Florence Viudes:** Investigation. **Céline Mahfouz:** Writing – review & editing, Visualization, Supervision, Methodology, Funding acquisition, Conceptualization. **Rachid Amara:** Writing – review & editing, Visualization, Supervision, Methodology, Funding acquisition, Conceptualization.

## Data availability

Data will be made available upon request.

## Funding

The authors are very grateful to 10.13039/100016592ULCO (University of Littoral Côte d’Opale) and the National Council for Scientific Research of Lebanon (CNRS-L) for financially supporting Rosa Sawan's PhD scholarship. This research is part of the TREASURE project (Targeting the reduction of plastic outflow into the North Sea) financially supported by Interreg North Sea programme project. It has also benefited from the grant "ANR-21-EXES-00 11″ as part of the IFSEA graduate school, which originates from National Research Agency under the Investments for the Future program.

## Declaration of competing interest

The authors declare that they have no known competing financial interests or personal relationships that could have appeared to influence the work reported in this paper.
