# Supplementary material for: Mobilization and deposition of plastic along an estuarine bank during tidal cycles
Source: Heliyon. 2025 Jan 21;11(2):e42026. doi: 10.1016/j.heliyon.2025.e42026 (PMC11804560; doi:10.1016/j.heliyon.2025.e42026)
Supplement: Multimedia component 1 [file mmc1.docx]

Supplementary data

Table 1. Overview of the conducted surveys

| Dates | June 16 – 17, 2022 | Sept 12-13, 2022 | Nov 24-25, 2022 | January 23-24, 2023 | April 20-21, 2023 | July 6-7, 2023 |
| --- | --- | --- | --- | --- | --- | --- |
| Tidal coefficient | 96 | 105 | 94 | 105 | 100 | 93 |
